# Oat Kilning and Its Effects on Liquid Oat-Base Production

**DOI:** 10.3390/foods13244083

**Published:** 2024-12-17

**Authors:** Sofie Ekelund, Eimantas Gladkauskas, Svenja Krause, Nick Sirijovski, Patrick Adlercreutz, Marilyn Rayner, Carl Grey, Cecilia Tullberg

**Affiliations:** 1 Division of Biotechnology, Lund University, Naturvetarvägen 16, SE-221 00 Lund, Sweden; 2 Global Oatly Science and Innovation Centre, Rydbergs torg 11, Space Building, Science Village, 224 84 Lund, Sweden

**Keywords:** oat processing, kilning, lipase activity, protein solubility, oat-base

## Abstract

The aim of this study was to give insights on the effects of an industrially relevant kilning method, with a focus on lipase inactivation and oat-base production. Storage of non-kilned, dehulled oat kernels in either room temperature or at 37 °C for up to 64 days led to increasing lipase activity with time, despite a decrease in moisture content and water activity, demonstrating the importance of kilning before storage. It was shown that the temperature and relative humidity used during the kilning had a major impact on both protein solubility and lipase inactivation. Steaming with saturated steam at 90 °C for 30 min followed by drying at 100 °C for 30 min was found to be enough to inactivate all lipases but still leave a relatively high protein solubility (42% of the total soluble protein). In the sensory trial, the indication was that kilning was a vital step from a sensory quality perspective, giving a pleasant aroma to the final oat-base product. However, with kilning, the final protein content decreased; thus, these results are important for the development of future oat-base products, where quality, nutritional content, and taste need to be balanced.

## 1. Introduction

Oats (*Avena sativa*) are cereal grains containing a lot of unsaturated fatty acids, proteins of high quality, starch, minerals, vitamins, and dietary fiber, such as β-glucan [1,2]. Oats are used both for human consumption as well as animal feed because of their nutritional content. Humans have mainly consumed the crop in forms of rolled oats, breakfast cereal, porridges, and flakes over the years, but lately there has also been a development of more liquid products, like yoghurts and oat milk [3,4,5]. Oats have further been connected to positive health benefits. Amongst others, it contains avenanthramides, molecules with antioxidative properties, which have been claimed to reduce inflammatory responses [6], and β-glucans, which contribute to lowering LDL cholesterol [7]. Oats have a relatively high protein content compared to other cereals, being around 17%, and furthermore have a good protein efficiency ratio of 1.8 compared to 0.8 in wheat [3]. Protein efficiency ratio is in turn a way of measuring the amino acid composition and digestibility of proteins, as a way to evaluate the protein quality of a food product [8].

There is, however, one main problem when it comes to oats. Compared to many other common cereals, oats have a high lipid content. The lipid content is, depending on the oat variety, at least 3.5 times higher in oats than in wheat [3], and the lipid content itself is often associated with the typical properties of oats, such as its pasting properties [9]. The lipids in oats can be hydrolyzed by lipases and are further prone to oxidation, which can result in rancidity and reduced shelf life and quality of the oats [1,9,10]. Furthermore, the lipolytic enzymes in oats have a ten to fifteen times higher activity, compared to those in wheat [11]. This combination of properties creates a challenge for the food industry, as it is of interest to prevent the development of off-flavor compounds as well as to keep the oat lipids stable [9]. 

What is commonly done to prevent lipid degradation is to treat the oats with a combination of steam and heat, in order to both inactivate enzymes in the outer bran layers as well as endogenous enzymes [1,9,12]. The treatment itself is known as kilning, and during the process, different parameters are of importance, such as temperature, time, and water content, all of which affect the final properties of the treated oats [4,9]. Besides inactivating enzymes, the heat treatment of oats can also lead to non-enzymatic oxidation, such as lipid autoxidation [10,13]. Oats have been said to have a relatively high oxidative stability due to their high antioxidant capacity, but the antioxidants can also be degraded as a result from the heat treatment [14,15,16]. Furthermore, the solubility of the proteins can also decrease due to processing, as the proteins might become denatured, which could affect their functional properties [5,17]. In a previous study, we saw a 20% decrease in soluble proteins in Swedish oats due to kilning [18]. In a study by Jaksics et al. (2023), heat treatment of oats had no effect on total protein content; however, an increase in molecular size distribution was observed, indicating protein aggregation [19]. The kilning process contributes to a change in flavor, which leads to nutty, toasted, and caramel-like flavors instead of the original hay-like flavor that comes naturally from oats [20]. This change in flavor is caused by Maillard reactions that are increased by the heat from the kilning and steam combined, creating reactions between proteins and carbohydrates, affecting both the flavor profile and color of the oats [10,21]. Finally, there is a strong connection between water activity and microbial growth, browning reactions, and lipid oxidation. If the water activity is too high, i.e., just below 1, the enzymatically induced oxidation rate increases; however, the autoxidation rate increases if the water activity is low [22]. An attractive approach would be to optimize the kilning process with respect to the minimal lipase activity for storage stability yet attractive flavor development; however, the oat lipase kinetics remain largely unexplored. 

Most functional characteristics of oats will be affected by its processing. There is currently considerable commercial interest in developing novel oat-base products from oats [20,23,24], especially in oat-based milk analogues, here referred to as liquid oat-base products. Liquid oat-base production can use different sources of oats, such as kilned whole oats, oat flour, or oat flakes, and involves wet milling, heating, enzyme treatment for optimal solubilization, viscosity and sweetness, and removal of insoluble particles (e.g., by centrifugation) [25]. For increased stability, a final pasteurization step can be included. Enzyme optimization for increased yield [26] and dietary fiber content [25] has been suggested as a suitable area for future development. A current difference between liquid oat-base products and milk is that they generally contain a low protein content as compared to milk [24,27,28]. In a study by Bonke et al. (2020), it was observed that heat treatment (65 °C, 45 min) combined with enzymatic treatment of the liquid oat-base products provided similar protein levels in the supernatant, compared to raw oat flour, i.e., 0.5% and 0.6%, respectively [29]. In the study, milled, and most likely kilned, oat flour was used (not stated). In a recent study by Liu et al. (2021), a modified milling step was further found to increase the final protein content of oat-base [30]. Much is yet to be investigated related to the effects of processing on the development of plant-based drinks, and, to our knowledge, no one has previously investigated the effects of kilning in liquid oat-base production. 

In this study, we aimed to evaluate an overall impact of kilning of oats on multiple aspects, affecting liquid oat-base production. The effect of temperature, time, and moisture, determined as water activities, during kilning was evaluated in small scale for different oat varieties and EMS mutants derived from the cultivar Belinda to optimize enzyme inactivation while keeping oxidation inducing heat at a minimum. Next, optimal kilning conditions were evaluated by protein solubility yield, enzyme deactivation, peroxide formation, and color, using settings mimicking industrial processing, with a variety of different oats. The oats kilned using the optimized conditions determined were further evaluated in a lab-scale liquid-oat-base-processing setting, where the impacts on selected parameters were investigated for increased stability and protein content in the final oat drink products. We show that by monitoring kilning, protein levels in the final liquid oat-base product can be increased, which is highly relevant for the food industry. 

## 2. Materials and Methods

### 2.1. Materials

#### 2.1.1. Chemicals

Cumene hydroperoxide (CPO), BaCl_2_, FeSO_4_, LiCl, ammonium thiocyanate, p-nitrophenol butyrate (pNPB), and bovine serum albumin (BSA) were purchased from Sigma Aldrich (St. Louis, MO, USA). The enzymes Amylase BAN 480L and Glucoamylase AMG from Novonesis (Copenhagen, Denmark) were used for oat-base creation. All solvents used were of HPLC grade or higher.

#### 2.1.2. Oats

For evaluation of kilning, whole oats of different varieties and EMS mutants derived from the cultivar Belinda from CropTailor AB, part of Lantmännen AB (Stockholm, Sweden), and samples from Kampetorp were kindly provided by Anders Jonsson (Swedish University of Agricultural Sciences). An in-house laboratory-scale Rivakka oat dehuller (Nipere Oy, Teuva, Finland), run at speed setting 7, were used, and the oats were allowed to pass three times. Oats kilned in the miniaturized oven were peeled by hand. For all other trials, dehulled oats of a commercial sample from the mill, both non-kilned and industrially kilned oats from the same batch, were used. The non-kilned oats had not been treated with any dry heat or steam, and the specific details of the industrially kilned oats were unknown. The in-house batch size between whole oats were in a similar range (25 kg); however, the field batches of the EMS mutants were considerably lower (<2 kg). Representative samples were aliquoted for use during the study. The industrially kilned oats were vacuum packed and kept in a cold room (4–6 °C) until further processing or analysis. All oat samples were frozen on the same day (−80 °C).

### 2.2. Storage

Mesh bags were prepared with non-kilned dehulled oat kernels and closed with zip ties (90 g). The bags were either placed on a glass Petri dish in a dark cupboard, or on in a controlled incubator (Labassco, Gallencamp Economy Incubator size 1) at 37 °C. A temperature and humidity logger (temperature and moisture logger with USB, ST171, Clas Ohlson) was placed in each storage location for up to 64 days. The data of the loggers were collected and reset after 32 days. The temperature and relative humidity levels in the incubator were, respectively, in range of 37.35 ± 0.65 °C and 19.25 ± 7.35% at day 0–32 and 37.45 ± 0.55 °C and 17.15 ± 8.65% at day 33–64. The logger set at room temperature conditions showed a variation of 21.1 ± 1.6 °C and relative humidity at 46.35 ± 9.95% at day 0–32, and at day 33–64, 20.55 ± 0.95 °C and relative humidity at 42.05 ± 16.95%. Samples were collected for lipase, color, and PV analysis throughout the time period.

### 2.3. Kilning

**Figure 1:**
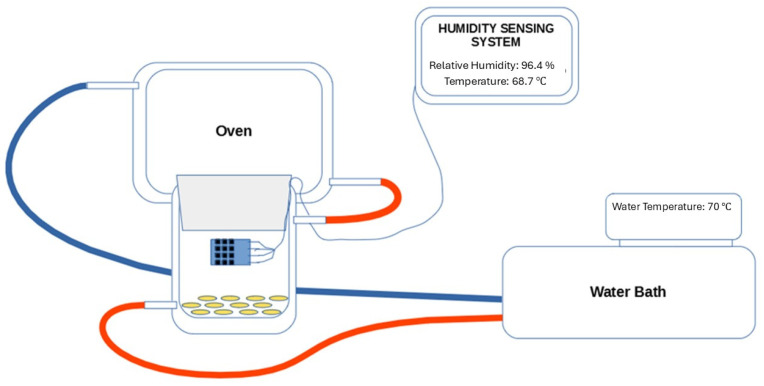
The portable miniaturized oven set-up designed to perform hydrothermal treatment of oats evaluating the kilning procedure.

Kilning was performed in a miniaturized oven set-up (150–250 mg oat), see schematic overview in Figure 1. A portable relative humidity system was attached and was designed to keep track of the relative humidity in the oven. A small single-board computer (Arduino NANO) was used as a microcontroller, and AM2320 relative humidity sensors (StyraHem, Luftfuktighet- och temperaturgivare, Aosong Electronics Co., Ltd., Guangzhou, China) were used inside the oven to keep track of the relative humidity and the temperature. The data were then retrieved through the application PuTTY. 

For all other kilning trials, a steam oven (SelfCookingCenter^®^ oven, Rational AG, Landsberg am Lech, Germany, 2017) was used, simulating industrial kilning. Dehulled oats (115 g) were put in a metallic sieve in the middle of the oven. The sieve was covered with perforated aluminium foil. The kilning was performed according to the settings in Table 1. An in-house system to log the humidity and temperature profiles were connected to the oven. A small computer (Arduino, UNO Wifi Rev 2) was used to log temperature and humidity through AM2302 and AM2320 sensors (StyraHem, Luftfuktighet- och temperaturgivare, Aosong Electronics Co., Ltd., Guangzhou, China). The data were then retrieved through the application PuTTY.

### 2.4. Determination of Water Activity 

Water activity (a_w_), defined as relative humidity at equilibrium divided by 100, is used as a quality variable connected to water migration and various food deterioration parameters [22]. The water activity of the samples was measured in triplicates directly after sample collection from the steam oven using a water activity meter (AquaLab Series 3, Decagon Devices, 2005, Pullman, WA, USA). The device was calibrated using a solution of LiCl (8.57 mol/kg in H_2_O, metergroup) with a known water activity of 0.500 before each run.

### 2.5. Determination of Moisture Content

The moisture content of the samples from the steam oven was measured in triplicates in a moisture analyzer (Mettler Toledo, HS153 Moisture Analyzer, Barcelona, Spain). All oat kernel samples were ground in a coffee grinder (Bodum, model 11160) and put on an aluminum plate (3 g) in the analyzer and dried at 105 °C until less than 1 mg of water evaporated for 50 s (constant mass).

### 2.6. Determination of Colour

#### 2.6.1. Spectrophotometric Measurement

The color change in the oat kernels was measured with a portable spectrophotometer using 15 replicates (CM-700d, Konica Minolta Sensing Inc., Osaka, Japan) with settings D_65_, corresponding to whiteness index WI (E313-96) i.e., standard illuminant daylight, color temperature 6504K as light source. The L*, a*, and b* values from each measurement were noted. The L* ranges from 0 to 100 and gives a value of lightness, and the a* and b* values range from negative to positive, giving values of the color spectra in red to green and yellow to blue coordinates, respectively [31]. From these data, amongst others, chroma (C*) can be calculated according to Equation (1), ranging from 0 to 181, where a higher value indicates a brighter color.

(1) $$C^{*}=\sqrt{{{(a}^{*})}^{2}+{{(b}^{*})}^{2}}$$

#### 2.6.2. RGB Analysis

Pictured were taken on each sample in a black photo box (Nikon D3300, AF-P DX 18-55/3.5-5.6G, Tokyo, Japan) and analyzed using the open-source software FIJI (release 2.16.0, including ImageJ, version 2.16.0) to obtain RGB histograms with R, G, and B values at 480K. Three smaller circles were marked in each photo to obtain analysis triplicates. 

### 2.7. Determination of Lipase Activity 

Lipase activity was measured in triplicates or more, using the pNPB method in a 96 well plate according to Pålsson et al. (2024) and in single cuvette for selected samples [32]. Freshly milled oat flour (100 mg) was dispersed in 1 mL sodium phosphate buffer (100 mM, pH 7.25), vortexed, centrifuged (1 min, 18,000× *g*), and the supernatant (45 μL) was added to a microwell plate together with sodium phosphate buffer (150 μL). To the substrate solution, 5 µL 20 mM pNPB was added, and the absorbance was immediately measured at 400 nm continuously for 5–15 min at 37 °C (ThermoScientific, Multiskan GO, Waltham, MA, USA). The molar absorption coefficient (ε) used for pNP at 400 nm and pH 7.25 was 16 775 M^−1^cm^−1^ [33]. For blanks, the oat extract was replaced with water. The pathlength for each well was set to 0.5677 cm. 

For single-cuvette analysis, a Shimadzu UV-1650PC spectrophotometer (Shimadzu, Duisburg, Germany) was used, and 100 µL of the oat supernatant solution prepared as above was added to 870 µL of the buffer, followed by 20 µL 20 mM pNPB solution. The cuvette was covered with foil and mixed via inversion. The absorbance was measured at 400 nm continuously for 2 min, and the pathlength of the cuvette was 1 cm. All other settings were the same as above.

### 2.8. Determination of Peroxide Value

Lipid extraction and peroxide value (PV) determination were performed according to Pålsson et al. (2024), with minor modifications described by Lindberg Yilmaz et al. (2021) and Undeland et al. (2002) [32,34,35]. Analyses were conducted in triplicates in clear glass vials with Teflon caps. Lipid extracts were kept at −80 °C prior to the analysis. Lipid extracts were obtained by adding 3.75 mL of chloroform/methanol (2:1 *v*/*v*, with 0.05% BHT *w*/*v*) to oats (200 mg), followed by 1 mL of 0.15 M acetic acid, 1.25 mL chloroform, and 1.25 mL water. The samples were vortexed between additions for 5 s, then centrifuged, and the lower chloroform phase containing the extracted lipids was collected. The samples were kept at –80 °C until analysis.

For PV determination, the samples were kept on ice in dark vials (4 mL) with Teflon caps. The lipid extract (500 µL) was mixed with chloroform (500 µL), followed by addition of methanol (1 mL). Ammonium thiocyanate solution (20 µL, 8 mg/mL) was added to each sample and vortexed (5 s). A freshly prepared Fe(II)CHCl_3_ solution was added (20 µL), and the samples were vortexed (5 s). The Fe(II)CHCl_3_ solution was prepared by dissolving FeSO_4_x7H_2_O (10 mg) in H_2_O (1 mL), adding a solution of BaCl_2_xH_2_O (1 mL, 8 mg/mL in 0.4 M HCl), then mixing, centrifuging, and collecting the supernatant. The samples were incubated for 20 min at room temperature, and the absorbance was measured at 500 nm in a glass cuvette (WPA, Biowave 3+, Biochrom Ltd., Cambridge, UK). Solutions (2 mL) of CPO ranging from 0 to 5 µM in 1:1 CHCl3:MeOH were prepared and used for the external standard curve. 

### 2.9. Determination of Protein Solubility 

Soluble protein was measured using the Bradford method in a 96-well plate. Oat kernels were milled into a flour with ≤0.5 mm particles (Laboratory mill 3100, Perten Instruments, Stockholm, Sweden), and 70 mg of flour was suspended in 1.5 mL of the sodium phosphate buffer (10 mM, pH 7) by vortexing for 10 s. Proteins were extracted using a thermoshaker (Eppendorf, ThermoMixer C, Hamburg, Germany) at 2000 rpm, room temperature, for 1 h. The samples were centrifuged at 3200 rpm for 10 min, and the supernatant (protein solution) was collected. In the 96-well plate, 5 µL of the protein solution was added in each well, followed by 250 µL of Coomassie blue reagent. The plate was incubated for 10 min at room temperature and the absorbance measured at 595 nm (Spark Microplate Reader, Tecan Group Ltd., Männedorf, Switzerland). At least triplicates were made for each sample. A standard curve was made using BSA at the concentrations of 0, 0.025, 0.125, 0.25, 0.5, 0.75, 1, 1.5, and 2 g/L.

### 2.10. Preparation of Oat-Base

Three different oat-bases were prepared based on non-kilned oats, oats kilned with the optimal program found in the kilning section (90 °C steaming), and industrially kilned oats. The oat-base was prepared in batches (*n* = 1) in a thermomixer (Vorwerk, Thermomix TM6), using 850 mL water preheated to 50 °C in the mixer, followed by the dispersion of 150 g freshly milled oat flour, and the temperature was increased to 70 °C. Thereafter, 0.45 g of Amylase BAN 480L was added, and the mixture was incubated for 20 min under gentle stirring. The oat slurry was then cooled to 55 °C in an ice bath and re-added to the thermomixer, followed by the addition of 0.3 g of Glucoamylase AMG for 5 min. The temperature was then increased to 95 °C and held for 20 min to inactivate the enzymes. The oat slurry was filtered through a 180 µm sieve, and the final oat base was collected and cooled. Sampling was performed before and after sieving, and samples before sieving were centrifuged at 3200 rpm for 10 min. 

The viscosity of the oat-bases was controlled using a Rapid Visco Analyzer (RVA4800, Perten instruments). For each batch, 25 mL of water, 4.41 g of flour, 11 µL of BAN, and 7 µL of AMG were used. High-pressure cannisters were used, and the two enzyme steps were divided into EZ1: the reaction of BAN, and EZ2: the reaction with AMG. The settings for each enzymation step were set to mimic the conditions in the thermomixer.

### 2.11. Determination of Total Protein of Oat-Base

The total protein content in the oat-base was determined using the AOAC 990.03 Dumas method [36]. Oat-base samples were frozen (−80 °C), freeze dried, then continuously stored at −80 °C until analysis. Freeze-dried flour (25 mg) was tin packed (3 mm) and placed in the autosampler tray (Flashea 1112 Series, Thermo Scientific). The packages were combusted at 950 °C with a constant supply of oxygen. The gases formed were carried by helium to a layer of copper at 840 °C for reduction. The water and carbon dioxide formed were trapped in columns. The nitrogen gas passed through to a GC, and the signal was detected through a thermal conductivity detector (TCD). The measured nitrogen content corresponded to a protein content (dry matter basis) using protein factors of 6.25 and 5.54. The protein conversion factor most commonly used is 6.25; however, this has been shown to be incorrect for cereal proteins, and instead, 5.54 has been suggested [37].

### 2.12. Sensory Evaluation

A sensory evaluation was performed on the three oat-bases by food scientists with experience in oat processing. The sensory evaluation was carried out by four participants: one male and three females. The tasting was conducted via blind evaluation where all participants had a score board where they could rank on a scale of 1–5 sweetness (low to high), grassiness (low to very hay like), milkiness (watery to creamy) and roastiness (not at all, to caramelized and round in flavor). Each participant could also write comments oat-base to describe the flavor. 

### 2.13. Statistics

For the majority of data, ANOVA tests were performed to determine statistical significance. An exception was lipase activity, where the Mann–Whitney test was used to compare the samples one by one, as equal variances could not be assumed. Experimental data from finding optimal kilning conditions using the miniaturized oven was fitted towards mathematical models using Python (version 3.7). Equation (2), was used to derive $k_{d}$:

(2) $$E=E_{0}*e^{-k_{d}*t}$$

where $k_{d}$ is the rate constant (*s*^−1^), E the enzyme activity, $E_{0}$ is the enzyme activity at time 0, t is time, and *E_a_* is the activation energy. All statistical testing were performed using XLSTAT, and a confidence interval of 95% was used (*p* < 0.05). If nothing else is stated, the number of replicates used for analysis was 3 or above.

## 3. Results

### 3.1. Effects of Storage 

#### 3.1.1. Moisture Content and Water Activity During Storage

The water activity and the moisture content decreased in the oat kernels with increasing storage time, as seen in Supplementary Materials, Figure S1. 

#### 3.1.2. Lipase Activity During Storage

**Figure 2:**
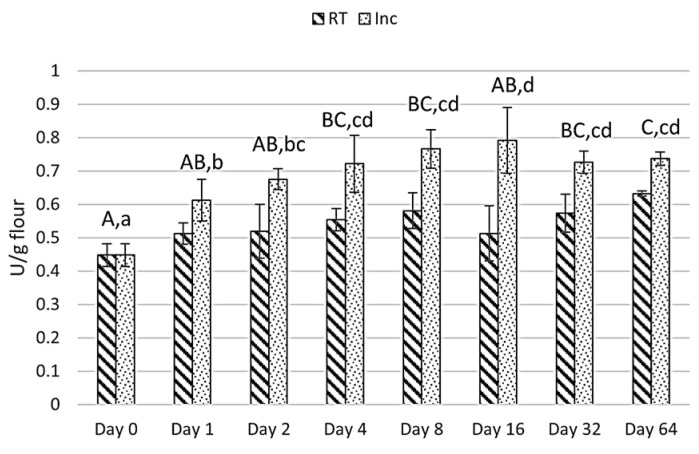
Lipase activity of the stored dehulled oat kernels from day 0 to day 64, please note that the data on the x-axis are not linear. Capital letters represent statistically significant differences (*p* < 0.05) for the room-temperature-stored oats (stripes) and lowercase letters for the oats incubated at 37 °C (dots). Standard deviations shown as error bars, *n* = 3.

A general increase in lipase activity with storage time was observed. The lipase activity during storage is shown in Figure 2.

#### 3.1.3. Color Change During Storage

No significant differences (*p* < 0.05) in color or image analysis could be seen when comparing the incubated and the room-temperature-stored samples after 64 days of storage (data not shown).

#### 3.1.4. Peroxide Value During Storage

PV analysis showed consistently very low values (<0.045 m.eq/kg oats), and no significant differences were found in the PV for any of the conditions tested.

### 3.2. Effects of Kilning

#### 3.2.1. Kinetic Study of Lipase Deactivation 

**Figure 3:**
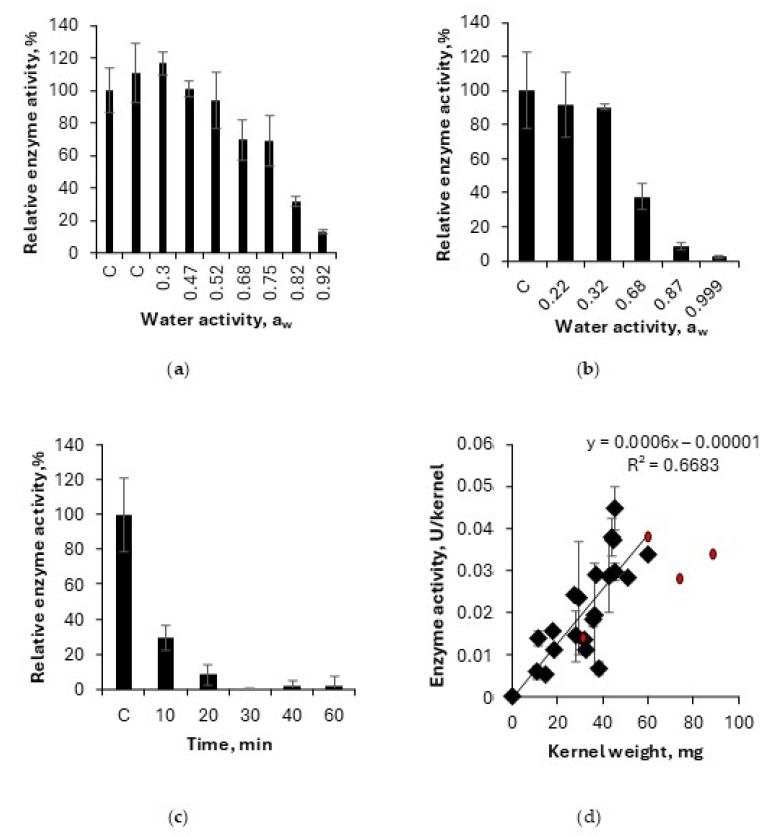
Influence of water activity, temperature, and time on oat lipase activity. Heat treatment was performed at different temperatures, 80 °C (**a**) and 90 °C (**b**), for 30 min with varying a_w_. Influence of time of oat lipase activity at 90 °C and 0.99 a_w_ (**c**). Lipase activity dependence on kernel weight, with red circles representing kernel weight including hulls (**d**). All other points in graph 3d are de-hulled oats. Letter “C” in graph 3a-c represents control samples, i.e., samples that were not heat treated. Error bars represent standard deviation (*n* = 3).

A decrease in lipase activity could generally be observed when the oat was kilned at different conditions. Evaluation of kilning was conducted using the miniaturized set-up. The effect of water activity during kilning on lipase activity is shown in Figure 3a,b, where samples were analyzed utilizing the single-cuvette pNPB method.

A kinetic trial investigating the time course of lipase inactivation was also conducted, and the results are shown in Figure 3c, where the temperature was 90 °C, 0.99 a_w_, and enzyme activity was monitored over 60 min. Enzyme activity dependence on the weight of kernel was investigated, and the results are shown in Figure 3d. The correlation between lipase activity and kernel weight using two kernels (*n* = 3) is further shown for the 17 different oat varieties in Supplementary Materials, Figure S2.

An additional set of experiments were conducted, where the impact of EtOH instead of water on lipase activity was investigated (data not shown). No significant effects were observed, where different amounts of EtOH were added to the oven during kilning. However, when pure EtOH (99%) was added (i.e., no water), it was seen that it was less efficient in deactivating the oat lipases compared to pure water.

**Figure 4:**
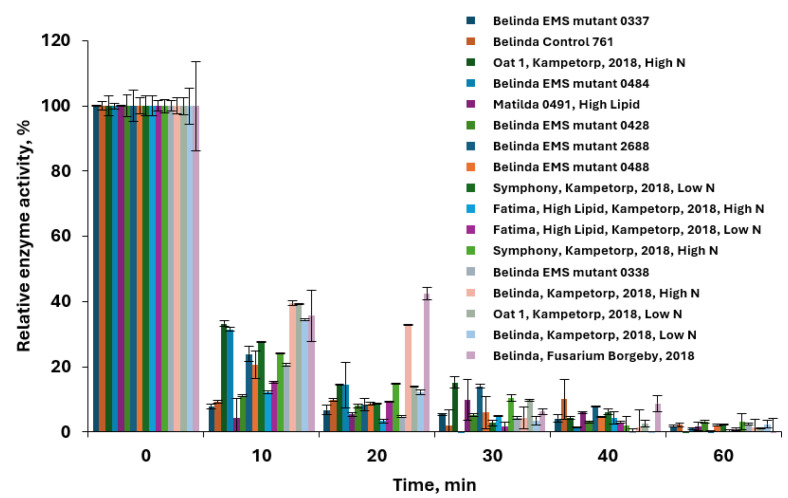
Hydrothermal treatment efficiency over time reducing the relative lipase activity. Treatment was conducted at 80 °C and 99.9% relative humidity. Seventeen varieties of oats were analyzed. Samples were analyzed using 96-well plate pNPB method (*n* = 3). EMS mutant corresponds to oats population developed by chemical modification from the variety Belinda, Oat 1 corresponds to oat with the annotation SW090315.

Next, the lipase activity was investigated for 17 selected oat varieties to evaluate potential genetic differences, and the results are presented in Figure 4. The lipase deactivation kinetics vary highly between different oat varieties and EMS mutants derived from the cultivar Belinda. The samples were run using the 96-well plate lipase assay; the temperature was set to 80 °C, the reaction was run at 0.99 a_w_, and the enzyme activity was monitored over 60 min. The rate constants were determined for the different oat varieties using Equation (2) and can be found in Supplementary Materials, Table S1. When modifying the temperature between 65 and 85 °C and following the kinetic behavior of the oats (data not shown), it was found that different oats were differently resistant to heat and had different lipase inactivation rate constants at different conditions.

The results from modelling are shown in Supplementary Materials, Figure S3. The data were fitted to Equation (2); however, they did not perfectly follow first-order kinetics. The data-fitting models investigated could not detect the difference between different activity inactivation kinetics. 

#### 3.2.2. Effect of Steam Oven Kilning on Lipase Activity

To better simulate an industrial-scale kilning, a steam oven was used. Overall, a higher temperature as well as a higher relative humidity during steaming led to a decrease in lipase activity. When the steaming temperature was altered, it was noted that a temperature of 90 °C was needed to completely inactivate all lipases. The obtained values can be seen in Table 2. 

When keeping the steaming temperature at 100 °C but changing the steaming humidity, it was noticed that at least 60% relative humidity was needed to completely inactivate the lipases. The experimentally measured humidity and corresponding lipase activity are shown in Table 3.

#### 3.2.3. Protein Solubility 

Processing might decrease protein solubility, as the proteins can become denatured, inducing protein aggregation and modification of the functional properties of the proteins. Protein solubility is therefore monitored. A clear decrease in protein solubility was seen for increasing treatment temperatures as well as increasing humidity shown in Table 4.

There was a marked difference between the industrially kilned oats that had the lowest solubility and the non-kilned oat flour that had the highest, with 0.172% and 2.17% soluble protein, respectively. As more severe kilning conditions decreased the protein solubility, it was decided to use 90 °C steaming at 100% RH for further oat-base processing, as this was deemed as the best conditions tested, resulting in a decent protein solubility and complete inactivation of the lipases in the oat-base.

#### 3.2.4. Moisture Content and Water Activity

Water activity is connected to moisture content, and both were monitored in this study. For oats, a water activity below 0.65 (13% in moisture content) is recommended to avoid microbial growth and high enzymatic activity [38]. For all kilning programs, the water activity and the moisture content were below 0.65 a_w_ and 11%, respectively, after kilning and drying step (100 °C, 0% RH, for 30 min), *n* = 3. 

#### 3.2.5. Color

The color change was measured in oats using a colorimeter, to evaluate if kilning induced any change in color, and acted as another food quality marker. No significant differences (*p* < 0.05) in color could be seen between the differently kilned samples. The non-kilned oats were found to be significantly different from all kilned oats in C* and b* value, as well as R value from the RGB-analysis. 

### 3.3. Oat-Base Results 

#### 3.3.1. Total Protein in the Oat Flour

The protein content in the non-kilned and industrially kilned oats is shown in Table 5, calculated with protein factor 5.54. The data show that the industrially kilned oat flour had a lower total protein content compared to the non-kilned (*p* = 0.006). The non-kilned flour is of the same oats as used in Table 4.

#### 3.3.2. Separation Method of Solids for Oat-Base Production

**Figure 5:**
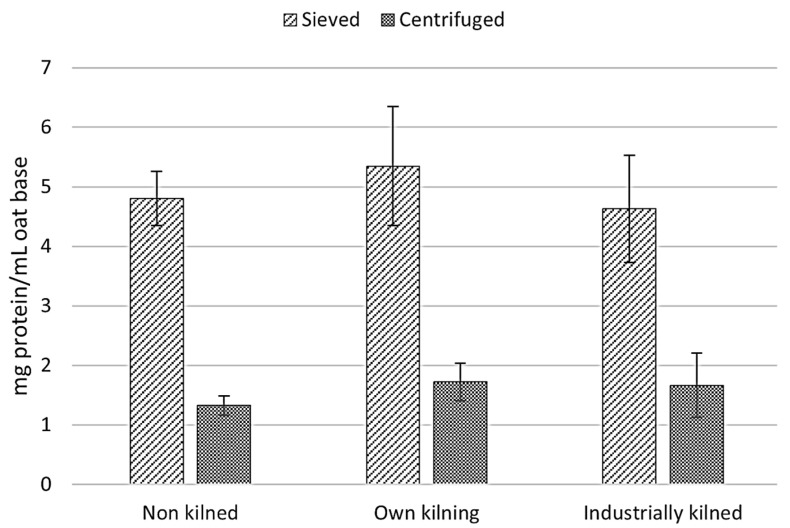
Protein content in the oat-bases processed with two different separation methods: sieving and centrifuging, measured by Bradford method. Standard deviations shown as error bars, *n* = 4.

To achieve the final liquid oat-base, a separation step is included where the fiber residue is separated from the oat base slurry. Here, sieving and centrifugation as separation methods were compared, and the protein content after the separation step was measured. Different protein content was obtained depending on if the oat-base slurries were sieved or centrifuged, see Figure 5. There were no significant differences found within the sieved samples, or within the centrifuged samples, but there was a significant difference (*p* < 0.05) between the different separation methods. 

#### 3.3.3. Moisture Content of the Oat-Bases

The moisture contents in the three oat-bases are shown in Table 6. They were found to be quite similar, but the in-house lab-scale kilning had a statistically higher dry matter content (*p* < 0.05). 

#### 3.3.4. Protein Content in the Oat-Bases

The total dry matter protein content (%) in the oat-bases in Table 7 were lower than for the pure flours, which is reasonable as some amount of oat that contains proteins is removed during the sieving of the oat-bases. It was seen that the industrially kilned oat-base had a significantly lower (*p* < 0.05) protein content than the other two oat-bases, but in general, there was not any huge difference. It should be noted that industrially kilned oat flour had an initially lower protein content compared to the non-kilned oats (Table 5), and that there is a clear difference in values obtained using the Bradford (Figure 5) or Dumas (Table 7) methods. Approximately 3-times higher values are obtained with the Dumas method. 

#### 3.3.5. Viscosity Measurements

In Table S2, the end-point viscosity values of the oat-base are shown, where high values are obtained when no enzyme is present, and low values are obtained with enzymes, as expected. Figures S4 and S5 in Supplementary Materials show the viscosity profiles for EZ1 and EZ2, mimicking a normal oat-base creation. Figure S6 shows EZ1 without enzyme, illustrating the importance of the enzymation steps for the viscosity of the final oat-base. The run with the EZ1 program without enzyme, seen in Figure S6, shows that the in-house kilning at 90 °C gave the highest oat-base viscosity. When looking at the data from Figures S4–S6, it can also be observed that the non-kilned oat-base had the highest viscosity after enzymatic treatment but the lowest viscosity when no enzyme was present. 

#### 3.3.6. Sensory Properties

A minor in-house sensory trial was conducted for comparison of the different oat-bases prepared. As the sample number was low, the data obtained from this investigation should be regarded as indicative. The oat-base made from non-kilned oat kernels tasted the most bitter and astringent according to the evaluation panel. This oat-base was perceived as the least favorable out of the three and was described as grassy in flavor. The oat-base made from industrially kilned oats tasted the most complex. It had more of the characteristic toasted and caramelized flavors that are usually present in commercial oat drinks. The in-house-kilned oat-base was perceived as the most neutral, as it was not as raw as the oat-base produced from non-kilned oats, nor as toasted in flavor as the oat-base produced from industrially kilned oats.

## 4. Discussion

### 4.1. Storage Stability of Dehulled, Non-Kilned Oats

The incubated, non-kilned, dehulled oats decreased faster in water activity and moisture content as compared to the room temperature stored samples, which was expected, as the higher temperature provided a higher driving force for the water to migrate from the kernels. A high moisture and water activity level is usually related to both high activity of enzymes and microbial growth. The water activity and the moisture content of the oat following the kilning programs were all below 0.65 a_w_ and 11%, respectively, indicating that there should not be problems of microbial growth [38]. However, for dry samples at low water activity (<0.25 a_w_), lipid oxidation is generally relatively high, due to the fact that the lipids have more access to oxygen when the sample is dry [39], although this might vary between different low-moisture foods. When the water activity reaches above 0.25 a_w_ or higher, the water becomes a barrier between the lipids and oxygen, and oxidation decreases. Thus, it would be likely that lipids in the incubated oat kernels (37 °C) were already more oxidized after 2 days of storage. This could not be confirmed by the PV data, however, due to low levels in all samples of this study. In this experiment, the surrounding environment was not controlled, but it was measured. Thus, it is expected that the moisture and water activity of the kernels would reach a plateau when the water in the kernel would be in equilibrium with the water in the air surrounding it, as long as the surrounding environment does not change. Between day 0 to day 32, the humidity in the surrounding air was quite stable. However, a dip in humidity was noticed between day 32 and day 64, which was caused by a decrease in outdoor temperature. This likely caused the change in the reduced moisture content and water activity observed in the oat kernels after day 32. 

According to Decker et al. (2014), too-high storage temperatures can lead to increased microbial growth as well as increased enzymatic reactions, which leads to a decrease in quality [38]. The lipase activity increased in the non-kilned, dehulled, incubated oats, with significant differences occurring already after 1 day. This indicates that enzymatic activity was initiated in these samples, both at room temperature and 37 °C. Once enzymatic activity is initiated, the shelf life of the oats is reduced, as free fatty acids are formed, leading to off-flavors. This shows the importance of storing the oats in a controlled environment and to perform kilning as close in time as possible to dehulling. There was also a trend showing an increase in lipase activity after longer storage, with a significant difference after 4 days at room temperature and after 1 day at 37 °C, despite the decreased water activity and moisture content. The higher lipase activity will contribute to formation of unwanted free fatty acids and other volatile compounds during the storage, which could affect the sensory properties of the final oat products. No color differences could be seen between the incubated oat kernels and the room-temperature-stored samples after 64 days of storage. This was expected, as no increase in PV was noticed, and as the storage temperatures were relatively low, no Maillard reactions, nor browning, were expected. This study indicates that it is very important to consider the treatment of the oats prior to kilning, as the storage trial showed increasing lipase activity following dehulling.

### 4.2. Effect of Kilning on Oats

#### 4.2.1. Lipase Inactivation Using Miniaturized Oven and Steam Oven

In this study, we show that there is a clear correlation between oat kernel size and lipase activity for whole, untreated oats; however, this was only true within a specific oat variety. The inherent enzyme activities differed more between different oat varieties, and when including the entire dataset, the correlation towards kernel weight was lost. The results from the kilning trials show that temperature, but most importantly the relative humidity, plays a pivotal role in deactivating the enzymes, and that most of the lipases are inactivated within 30 min (80–90 °C). The strongest effect was obtained at maximum a_w_, 0.99, as expected. This is in line with previous findings, where an increasing moisture content suppressed free fatty acid formation [40], and a steaming step in the kilning has been shown to inactivate lipase [10,13,41]. Furthermore, it was shown that despite the clear decrease in lipase activity at 80 °C, even lower levels were obtained at 90 °C. This can be compared with the study by Ekstrand et al. (1992), where oat immersed in water at 80 °C for 10 min resulted in 2% residual lipase activity [13]. Furthermore, it was seen that there are large variations in lipase activity when studying different oat varieties and EMS mutants derived from the cultivar Belinda. The optimal kilning setting might therefore vary, depending on which specific oat variety is used. The importance of water activity is related to the lubrication properties of water, which makes the enzymes more flexible, crucial for their catalytic function, but also increases the rate of their inactivation [42,43]. The other effect that is involved in the kilning process is heat transfer. At a higher relative humidity, the heat is transferred more efficiently, thus increasing the deactivation of the enzymes [40]. When simulating an industrial kiln, using an in-house steam oven, focusing on steaming humidity, it was noticed that at least 60% humidity was needed at 100 °C to inactivate lipases; however, when focusing on steaming temperature, it was noted that a temperature of 90 °C was needed to inactivate all lipases. The steam oven is, however, less controlled compared to the miniaturized oven. The miniaturized oven developed in this study was proven to be an excellent way to control the humidity and temperature of the system in detail, whereas there is less control of temperature and humidity in the pilot steam oven, and even less so in an industrial kiln. For mechanistic studies, including kinetic evaluation of endogenous enzymes, this set-up provides valuable data that are difficult to obtain in the less controlled environments. The lipase inactivation did not follow simple first-order kinetics. It is highly probable that most of the samples contain more than one lipase, which are inactivated with different rates, having different inactivation constants. Modelling lipase activity kinetics during kilning using Equation (2) showed a fairly good fit towards experimental data from the miniaturized oven; however, more work in this area is needed for good prediction of enzyme behavior, especially connected to different oat varieties. 

#### 4.2.2. Effect of Kilning on Protein Solubility

A highly relevant question for the development of oat-based products is the effect of kilning on protein content in the final product. A clear decrease in soluble protein could be seen for increasing kilning temperatures as well as increasing humidity, indicating that the kilning is a crucial step to control. This indicates that a harsh kilning process decreases the protein content in oat-base products. The industrially kilned oats had the lowest solubility, showing that the industrial kilning step is even more harsh in comparison with the in-house treatment. From the non-kilned oat flour, we obtain the highest content of 2.17% of soluble protein. This extraction was conducted in aqueous media with low salinity, and considering that oat has been reported to have around 17% in protein [15] and that mainly the albumins (9–20% of total protein) are extractable in water [44], this result is in line with expected data. Heat treatment (90–100 °C) was however found to reduce the relative protein content in the oat flour by 58–84.5%, compared to non-kilned oats. In previous studies, a 48% (Kerstin) [5] and 20% (Galant, Fatima, and Belinda) [18] reduction in the total amount of soluble protein was seen in the heat-treated samples, compared to non-kilned oats. It has been argued that differences might be due to specific oat varieties. In this study, the soluble protein content after the 90 °C steaming (100% RH) was higher than the soluble protein content of the 100 °C steaming at 60% RH. 

We did not expect to see much difference in total protein between the non-kilned and industrially kilned oats, but although not substantial, there was a significant difference. The industrially kilned oats were all from the same batch, but it is possible that this difference was due to additional cleaning and separation steps before the kilning itself, which could have removed some of the proteins, e.g., if damaged and low-weight kernels were removed. It is hypothesized that higher weight kernels have a higher percentage of starch content, leading to a slightly lower protein percentage. 

### 4.3. Oat-Base Production

The dry matter of the oat flours used differed slightly. The non-kilned, in-house and industrially kilned flour had a dry matter content of 88.2%, 92.1%, and 87.8%, respectively. This could explain why the in-house-kilned oat-base was highest in viscosity, as a constant quantity of oat flour was used. For both enzymation steps, EZ1 and EZ2, the non-kilned oat-base had the highest viscosity, indicating a higher internal resistance. The two kilned oat-bases were quite similar in viscosity, but the industrial one was a bit lower. This could be a result of the heat treatment making the starch in the oats more accessible for the enzymes, leading to a more extensive starch hydrolysis. Enzymatic treatment is needed to decrease the viscosity of the oat-base, as can be seen in Figures S4–S6 in Supplementary Materials, as expected. A strong gelling effect of oat starch in oat-base has previously been reported by, e.g., Bonke et al. (2020) [29]. 

A big difference in protein content was obtained depending on if the oat-base slurries were sieved or centrifuged. One hypothesis for this is that non-soluble proteins are spun down during the centrifugation, whereas most of them pass through the sieve and are still present in the oat-base. Others have suggested that insoluble compounds could be kept from sedimenting thanks to an amylopectin and amylose gel-network carrying them, and much of the protein could be kept in solution even after the enzymatic step [29]. The enzymation step is of high importance for the rheological properties of the oat-base, and the enzymes used in this study were very effective. 

All oat-bases were heated at 95 °C for 20 min for enzyme inactivation, which will most likely also affect the proteins. As the sieving takes place after the heating, there is a large risk that more of the protein aggregates and denatured proteins are removed in the fiber residue, thus mitigating the effect of performing a mild kilning, or even no kilning to improve the protein content. It would therefore be interesting to look further into alternative processing routes for oat-base, for example, where the product is sieved before the heat treatment stage. 

The dry matter content of the oat-bases varied between 13.6 and 14.5%. This was considerably higher compared to the study by Silventoinen-Veijalainen et al. (2024), where the dry matter content was 9.4% on average [27]. The oat-bases contained 1.5–1.7 g proteins/100 g oat-base when measured using Dumas, which can be compared to 0.7 g/100 g protein detected in a commercially available organic oat drink by Oatly [45], or 0.63–1.10% protein in a recent study investigating the impact of oat flour characteristics on oat-base [27]. This might be due to the specific set-up connected to removal of solid particles used in the different studies, where we used a sieve (Ø 180 µm), compared to Silventoinen-Veijalainen et al. (2024) who had both a centrifugation step and a sieve (0.8 mm × 0.8 mm) [27]. The two different methods for measuring protein (Dumas and Bradford) gave significantly different results, in line with previously reported data for plant-based drinks [46]. While Dumas is based on total nitrogen content, Bradford is a simpler spectrophotometric method, relying on soluble protein binding to Coomassie dye and forming a chromophore [46]. Bradford is therefore easily subjected to interference from surrounding molecules, protein aggregation and similar and will only reflect levels of soluble proteins [46]. Bradford gave in this study protein values nearly three times lower than those of Dumas. The oat-base samples were perceived as milky even when diluted, so it is likely that the protein content was underestimated in the Bradford analysis. Furthermore, the results obtained by the Bradford and the Dumas methods indicate a low level of soluble protein content of the oat-base. 

Our findings suggest that it is more advisable to use saturated steaming, but at lower temperatures during the kilning of oats to be used for oat-based products, as this leads to both inactivation of enzymes as well as a higher amount of soluble protein. However, heat treatment of the liquid oat-base to deactivate added enzymes will also affect the soluble protein content in the final product, and it might be desirable to delay this inactivation step to the already packaged product. This needs to be further evaluated; however, it could be one way to maintain more of the proteins in the final product. Furthermore, our results indicate that the processing steps of the oat-base products have a high impact of the final protein content, especially during the separation step where the fiber residue is separated from the oat-base slurry, and a clear difference in protein content was observed between sieving and centrifugation. These results could be of particular interest if a higher protein product is desired.

### 4.4. Sensory Aspects

The oat-base made from non-kilned oat kernels had the most bitter and astringent taste according to the evaluation panel. This could be due to a higher amount of polyphenolic compounds, such as avenanthramides or ferulic acids [20]. This oat-base was perceived as the least favorable flavor out of the three and was described as grassy. The oat-base made from industrially kilned oats had the most complex taste. Its taste was characterized as toasted and caramelized, which is common in commercial oat drinks. This is most likely due to Maillard reactions taking place during the harsher industrial kilning conditions. The in-house-kilned oat-base was perceived as the most neutral, as it was not as raw or as toasted in flavor. Worth mentioning is that there are usually more steps in an industrial oat-base production as compared to a small batch lab-scale oat-base, which could contribute to the sensory properties of the investigated products as well. To improve taste, it would be possible to perform steam flashing, which could remove some of the volatile grassiness and bitter flavors present. 

Overall, this study shows the importance of the kilning step affecting the properties of the final product, including important sensory characteristics. Many commercial oat-based product manufacturers purchase oats that have already been kilned, either in the form of flour or as whole, dehulled kernels. As a result, they have little influence over the kilning and dehulling processes. Industrially kilned oats had the lowest protein solubility, indicating harsh processing conditions. The purpose is probably to thoroughly inactivate enzymes and to increase Maillard reactions, i.e., caramelization and flavor development. Even though the industrially kilned oat-base was more similar to a commercial oat drink in the sensory aspects, it could be interesting for the future to continue investigating a milder processing of oats to create more neutral and more high protein products. A milder kilning process will, however, most likely affect the sensory aspects, where increased kilning commonly contributes with attractive flavor profile. 

## 5. Conclusions

This study highlights significant factors influencing the quality of oat-based products, focusing primarily on the connection between enzyme activity, soluble proteins, and processing conditions. While kernel size correlated with lipase activity within specific oat varieties, this relationship did not hold across different oat varieties, indicating that certain oat varieties might be more suitable from a processing perspective. Pre-kilning treatments, like dehulling, had a clear effect on lipase activity in the oats, and settings of the kilning parameters greatly impacted functional and sensory attributes of the oat-based products. Harsh industrial kilning inactivates enzymes but reduces protein solubility, whereas milder kilning methods led to preserved protein content of the final oat-based drinks. Adjusting kilning processing techniques, such as saturated steaming at lower temperatures, may balance enzyme inactivation with protein retention. Furthermore, post-kilning processing, such as sieving versus centrifugation, significantly influenced protein content. These findings suggest opportunities for producing oat-based products with higher protein content and tailored flavor profiles. However, future research is needed to refine oat processing methods, including kilning, to optimize the stability, as well as nutritional and sensory properties of the final products.

## Figures and Tables

**Table 1:** The kilning conditions used on dehulled oat kernels with steaming for 30 min each. A drying step was added, using 100 °C, 0% RH, for 30 min for all treatments.

Treatment Name	Steaming (30 min)
60 °C steaming temperature	60 °C, 100% RH
70 °C steaming temperature	70 °C, 100% RH
80 °C steaming temperature	80 °C, 100% RH
85 °C steaming temperature	85 °C, 100% RH
90 °C steaming temperature	90 °C, 100% RH
100 °C steaming temperature and RH ^1^ humidity	100 °C, 100% RH
110 °C steaming temperature	110 °C, 100% RH
120 °C steaming temperature	120 °C, 100% RH
40% RH ^1^ steaming humidity	100 °C, 40% RH
60% RH ^1^ steaming humidity	100 °C, 60% RH

^1^ Measured relative humidity during steaming.

**Table 2:** Lipase activity for different kilning temperatures. All oats were steamed for 30 min at different temperatures and saturated steam (100% RH), followed by a drying step at 100 °C for 30 min (0% RH). n.d. stands for non-detectable activity, and statistical letter represents if there is a significant difference (*p* < 0.05) between the samples according to a Mann–Whitney test, *n* = 3.

Kilning Temperature	Lipase Activity (Units/g Flour)	Statistical Letter (Mann–Whitney)
Non-kilned	0.379 ± 0.081	A
60 °C	0.113 ± 0.081	B
70 °C	0.026 ± 0.003	C
80 °C	0.008 ± 0.002	D
85 °C	0.003 ± 0.001	E
90 °C	n.d.	F
100 °C	n.d.	F
Industrially kilned	n.d.	F

**Table 3:** Lipase activity for different kilning conditions with varying relative humidity. All oats were steamed for 30 min at 100 °C and different relative humidities (40–100% RH), followed by a drying step at 100 °C for 30 min (0% RH). n.d. stands for non-detectable activity, and statistical letter represents if there is a significant difference (*p* < 0.05) between the samples according to the Mann–Whitney test, *n* = 3.

Kilning Humidity	Lipase Activity (Units/g Flour)	Statistical Letter (Mann–Whitney)
Non-kilned	0.379 ± 0.081	A
40% RH	0.018 ± 0.009	B
60% RH	n.d.	C
100% RH	n.d.	C
Industrially kilned	n.d.	C

**Table 4:** Protein solubility determined with the Bradford method for buffer extracted kilned flours processed using the different kilning programs, 30 min each. A drying step was added, using 100 °C, 0% RH, for 30 min for all treatments. Data shown in both protein solubility and relative protein solubility (*n* = 4) with standard deviations. Statistical letters indicate significant differences between samples (*p* < 0.05) using ANOVA.

Kilning Program	Protein Solubility % (mg Protein/mg Oats × 100)	Relative Protein Solubility (% of Non-Kilned Flour Solubility)	Statistical Letter (ANOVA)
Non-kilned	2.17 ± 0.03	100 ± 1.5	A
60 °C steaming temperature	1.72 ± 0.02	79.4 ± 0.77	B
70 °C steaming temperature	1.64 ± 0.06	75.6 ± 2.7	B
80 °C steaming temperature	1.17 ± 0.06	53.8 ± 2.8	C
40% RH ^1^ steaming humidity	1.23 ± 0.03	56.7 ± 1.5	C
90 °C steaming temperature	0.914 ± 0.09	42.2 ± 4.2	D
60% RH ^1^ steaming humidity	0.772 ± 0.06	35.6 ± 2.5	D
100 °C steaming temperature and 100% RH ^1^ humidity	0.336 ± 0.02	15.5 ± 1.1	E
Industrially kilned	0.172 ± 0.07	7.95 ± 3.3	F

^1^ Measured relative humidity during steaming, 100 °C.

**Table 5:** Total protein content of non-kilned and industrially kilned flour as percent (*w*/*w*) per dry weight calculated from the protein factor 5.54, determined by the Dumas method. Results shown with standard deviations, *n* = 3. Statistical letters indicate significant differences between samples (*p* = 0.006) using two-tailed *t*-test assuming unequal variances.

Milled Oat Flour	Protein %	Statistical Letter (*t*-Test)
Non-kilned	13.4 ± 0.12	A
Industrially kilned	12.8 ± 0.074	B

**Table 6:** Moisture content in the three oat-bases created from non-kilned, the in-house lab-scale kilned (90 °C steaming), and industrially kilned oats. Oat-base was sieved to remove solids prior to measurement. Results shown with standard deviations, *n* = 3. Statistical letters indicate significant differences between samples (*p* < 0.05) using ANOVA and two-tailed *t*-test assuming unequal variances.

Oat-Base	Moisture Content %	Statistical Letter (ANOVA)
Non-kilned	86.2 ± 0.16	A
In-house kilning	85.5 ± 0.0058	B
Industrially kilned	86.4 ± 0.17	A

**Table 7:** Protein content in mg/mL in the oat-bases and as percent (*w*/*w*) per dry weight oat-base after sieving: from non-kilned, the lab-scale in-house-kilned oats (90 °C steaming) and industrially kilned oats. Protein% corresponds to values calculated using protein conversion factor 5.54, measured using the Dumas method. *n* = 3, standard deviations included. Statistical letters indicate significant differences between samples (*p* < 0.05) using ANOVA and two-tailed *t*-test assuming unequal variances.

Oat-Base	Protein %	mg Protein/mL Oat-Base	Statistical Letter (ANOVA)
Non-kilned	12.2 ± 0.21	16.9 ± 0.30	A
In-house kilning	12.0 ± 0.26	17.4 ± 0.38	A
Industrially kilned	11.4 ±0.040	15.4 ± 0.054	B

## Data Availability

The original contributions presented in this study are included in the article/supplementary material. Further inquiries can be directed to the corresponding author.

## Supplementary Materials

The following supporting information can be downloaded at: https://www.mdpi.com/article/10.3390/foods13244083/s1, Figure S1: Effects on moisture content and water activity on the stored dehulled oat kernels from day 0 to day 64: (a) moisture content (%); (b) water activity. Standard deviations shown as error bars, n = 3; Figure S2: An enzyme activity dependence on a two kernel samples (n = 3). It can be seen that samples with very different weights can have very similar activity. Samples were analyzed using 96-well plate p-NPB method. EMS mutant corresponds to oats population developed by chemical modification from the variety Belinda, Oat 1 corresponds to oat with the annotation SW090315; Figure S3: Evaluation of modelling using Equation (2). Experimental data came from experiment conducted at 80 °C, 99.9% relative humidity at varied times. The decay of enzyme activity was measured using p-NPB method; Figure S4: The first enzymation, EZ1, of the three made oat base batches. The oat bases were made from non-kilned, the in-house lab-scale kilned (90 °C steaming), and industrially kilned oats. Lines represent one single replicate; Figure S5: The second enzymation, EZ2 of the three oat base batches. The oat bases were made from non-kilned, the in-house lab-scale kilned oats (90 °C steaming), and industrially kilned oats. Lines represents one single replicate; Figure S6: The oat base batches run with EZ1 as program, but without enzyme, showing the gelling properties of the starch in the differently kilned flours. Lines represents one single replicate. The oat bases were made from non-kilned, the in-house lab-scale kilned (90 °C steaming), and industrially kilned oats; Table S1: Lipase activity and lipase inactivation rate constants kd in different varieties of oats at different temperatures, calculated by Equation (2). EMS mutant corresponds to oats population developed by chemical modification; Table S2: The final viscosity obtained from the RVA batches of liquid oat-base development, both with and without enzyme. EZ1 represents the temperature profile for the first enzymation using BAN 480L, and EZ2 represents the heating profile for the second enzymation using AMG.
