# Supplementary material for: Oat Kilning and Its Effects on Liquid Oat-Base Production
Source: Foods. 2024 Dec 17;13(24):4083. doi: 10.3390/foods13244083 (PMC11675130; doi:10.3390/foods13244083)
Supplement: Supplementary file 1 [file foods-13-04083-s001.zip › foods-3333296-supplementary.pdf]

Supplementary Materials

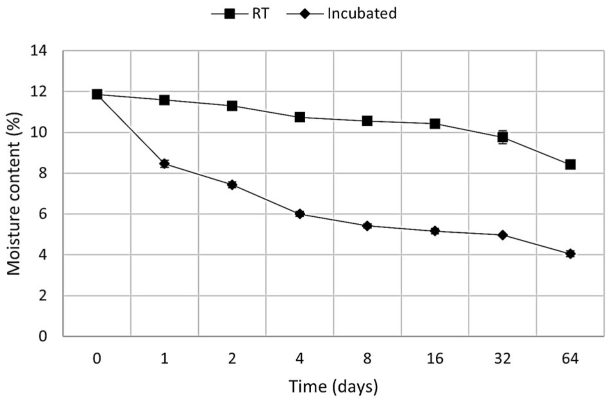

(**a**)

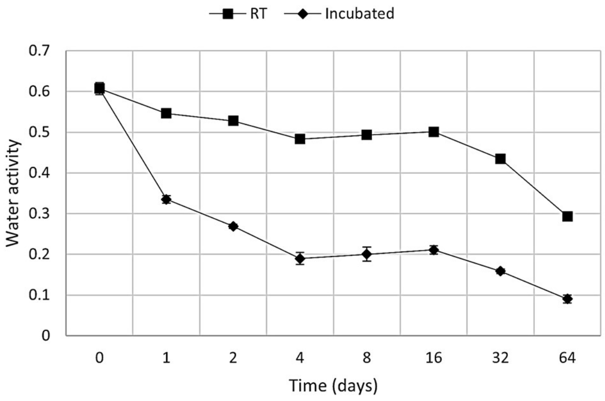

(**b**)

Figure S1. Effects on moisture content and water activity on the stored dehulled oat kernels from day 0 to day 64: (**a**) moisture content (%); (**b**) water activity. Standard deviations shown as error bars, $n = 3$.

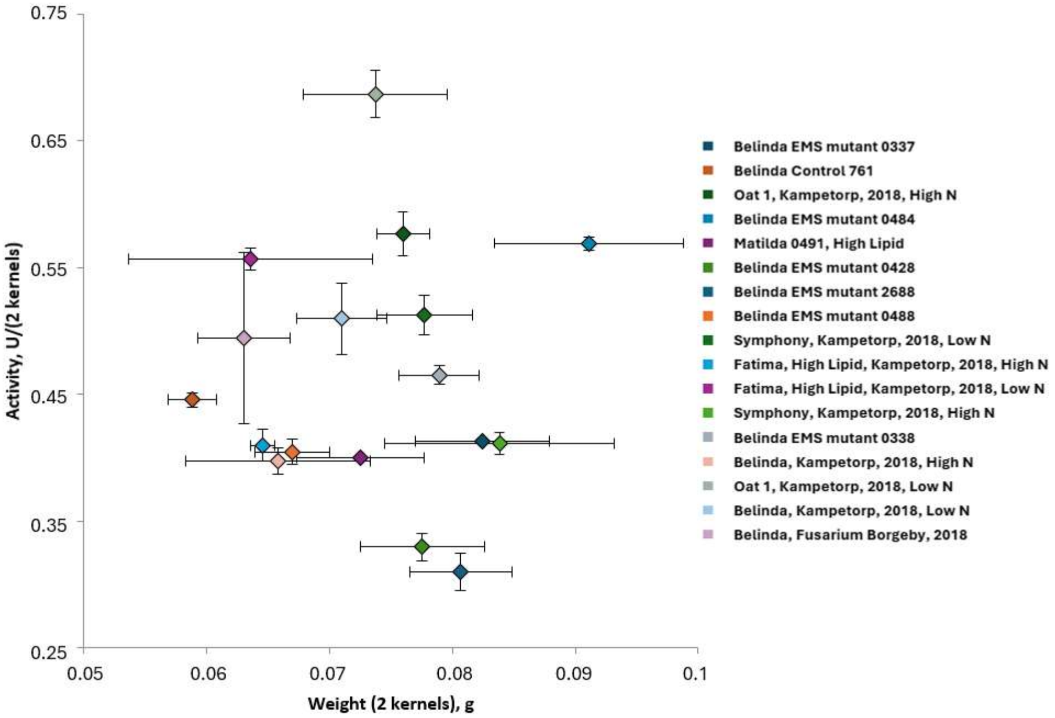

Figure S2. An enzyme activity dependence on a two kernel samples ($n = 3$). It can be seen that samples with very different weights can have very similar activity. Samples were analyzed using 96-well plate p-NPB method. EMS mutant corresponds to oats population developed by chemical modification from the variety Belinda, Oat 1 corresponds to oat with the annotation SW090315.

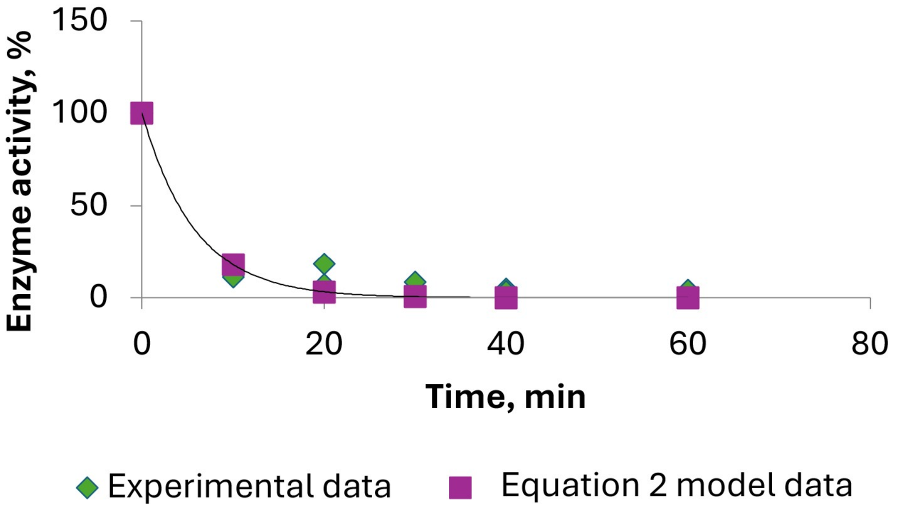

Figure S3. Evaluation of modelling using Equation (2). Experimental data came from experiment conducted at 80 °C, 99.9% relative humidity at varied times. The decay of enzyme activity was measured using *p*-NPB method.

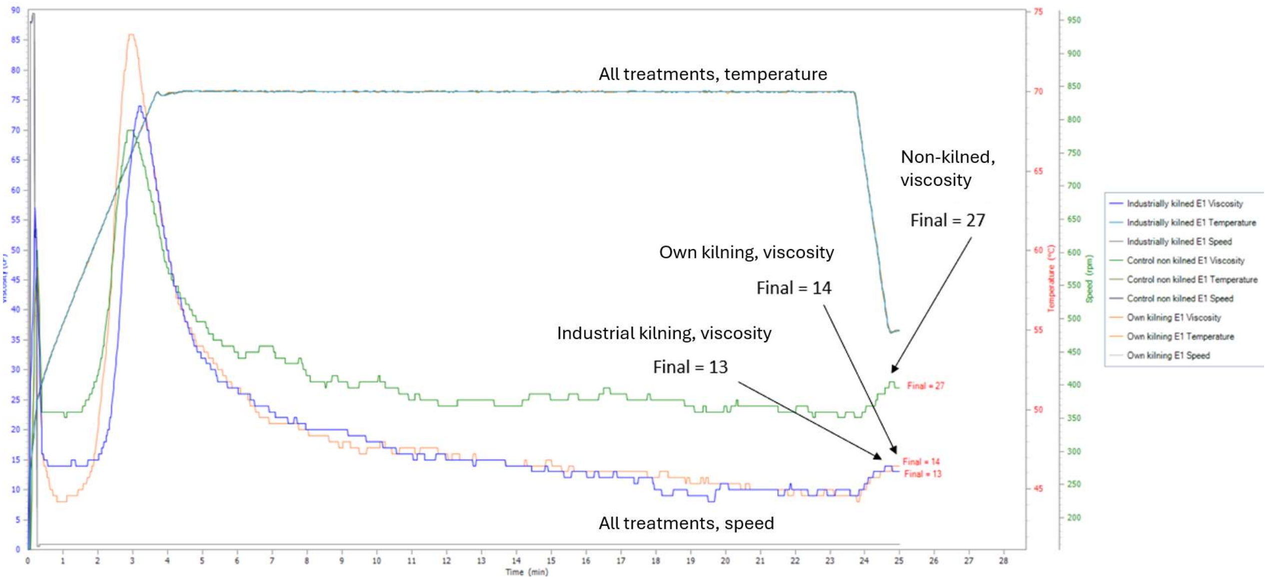

Figure S4. The first enzymation, EZ1, of the three made oat base batches. The oat bases were made from non-kilned, the in-house lab-scale kilned (90 °C steaming), and industrially kilned oats. Lines represent one single replicate.

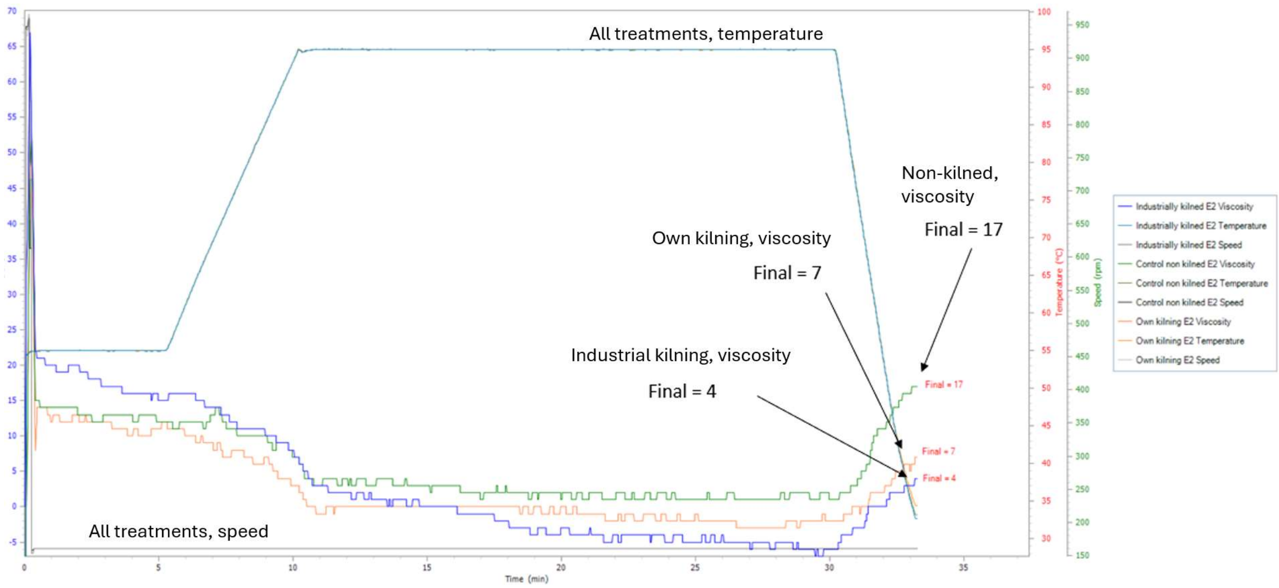

Figure S5. The second enzymation, EZ2 of the three oat base batches. The oat bases were made from non-kilned, the in-house lab-scale kilned oats (90 °C steaming), and industrially kilned oats. Lines represents one single replicate.

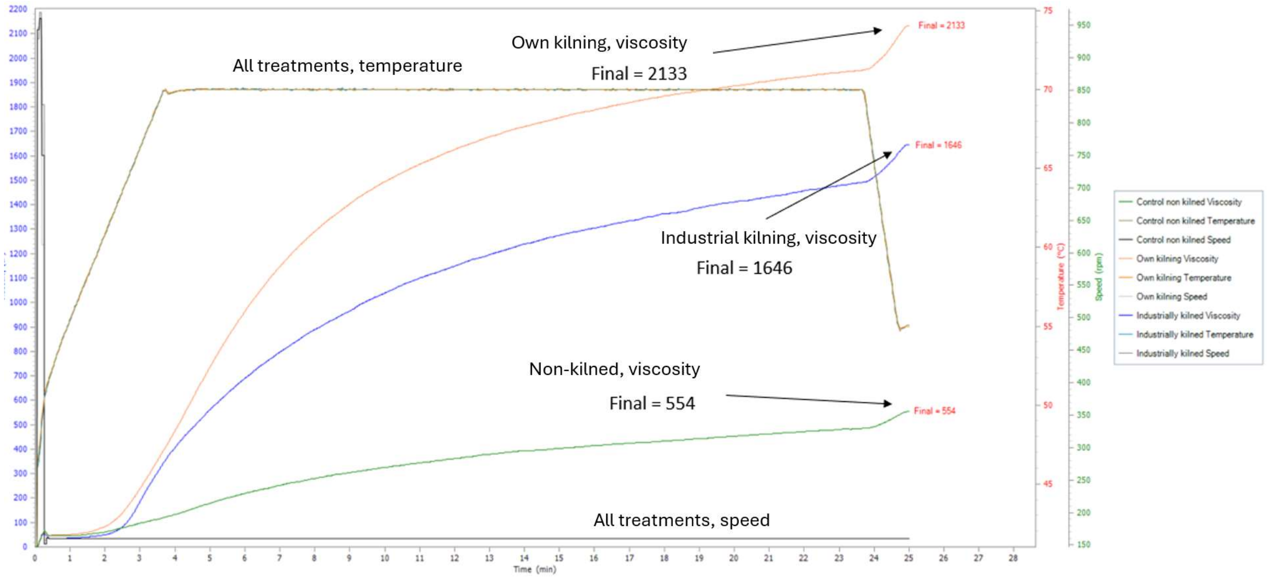

Figure S6. The oat base batches run with EZ1 as program, but without enzyme, showing the gelling properties of the starch in the differently kilned flours. Lines represents one single replicate. The oat bases were made from non-kilned, the in-house lab-scale kilned (90 °C steaming), and industrially kilned oats.

Table S1. Lipase activity and lipase inactivation rate constants k_d in different varieties of oats at different temperatures, calculated by Equation (2). EMS mutant corresponds to oats population developed by chemical modification.

Variety	Phenotype and Trial	Lipase Activity, U/g	k_d 85 °C	k_d 80 °C	k_d 70 °C	k_d 65 °C
Belinda	0337, field-grown	5.558	1.882	0.236	0.022	0.017
Belinda Control	761, field-grown	6.329	-	0.615	0.059	-
Matilda	High lipid, field-grown	5.689	-	0.427	0.040	-
EMS mutant derived from Belinda	2688, expected high protein, field-grown	5.265	-	0.361	0.034	-
Fatima	High lipid, field-grown	5.981	-	0.443	0.042	-
Belinda	Fusarium, Borgeby 2018, field-grown	6.698	-	0.098	0.037	-

Table S2. The final viscosity obtained from the RVA batches of liquid oat-base development, both with and without enzyme. EZ1 represents the temperature profile for the first enzymation using BAN 480L, and EZ2 represents the heating profile for the second enzymation using AMG.

RVA Program	With/Without Enzyme	Oat Flour	Final Viscosity (cp)
EZ1	With	Non-kilned	27
		In-house kilning	14
		Industrially kilned	13
EZ2	With	Non-kilned	17
		In-house kilning	7
		Industrially kilned	4
EZ1	Without	Non-kilned	554
		In-house kilning	2133
		Industrially kilned	1646
